# 284. Age-related differences in B-cell response during *Clostridioides difficile* infection in mice

**DOI:** 10.1093/ofid/ofad500.356

**Published:** 2023-11-27

**Authors:** Archit Kumar, Martin O’Brien, Nhu Nguyen, Kimberly Vendrov, Ingrid L Bergin, Vincent B Young, Raymond Yung

**Affiliations:** Department of Internal Medicine, Division of Geriatric and Palliative Medicine, University of Michigan, Ann Arbor, MI, Ann Arbor, Michigan; Department of Internal Medicine, Division of Geriatric and Palliative Medicine, University of Michigan, Ann Arbor, MI, Ann Arbor, Michigan; Department of Internal Medicine, Division of Infectious Diseases, AND Department of Microbiology and Immunology, University of Michigan, Ann Arbor, MI., Ann Arbor, Michigan; Department of Internal Medicine, Infectious Diseases Division University of Michigan, Ann Arbor, Michigan, Ann Arbor, MI; Unit of Laboratory Animal Medicine, University of Michigan, Ann Arbor, MI, Ann Arbor, Michigan; University of Michigan, Ann Arbor, MI; Department of Internal Medicine, Division of Geriatric and Palliative Medicine, University of Michigan, Ann Arbor, MI, Ann Arbor, Michigan

## Abstract

**Background:**

*Clostridioides difficile* (*C. difficile*) infection (CDI) is a leading cause of nosocomial infection that can range from mild diarrhea to life threatening pseudomembranous colitis and death. Aging as well as defects in the host immune response including failure to develop antibody response against *C. difficile* antigens increases the risk of bacterial colonization, hospitalization, mortality and developing recurrent CDI. The present study aimed to investigate the differences in the B cell response between young and aged *C. difficile* infected mice.

**Methods:**

C57BL/6 mice were rendered susceptible to CDI via the administration of oral cefoperazone for 10 days and then the mice were challenged with 10^3^ spores of *C. difficile* R20291 strain (Figure 1). The young (3-4 months) and aged (22-24 months) mice were euthanized at 14 days post infection (dpi). The germinal center response and IgA+ antibody-secreting cells (ASCs) were studied using multicolor flow cytometry.

**Figure:**
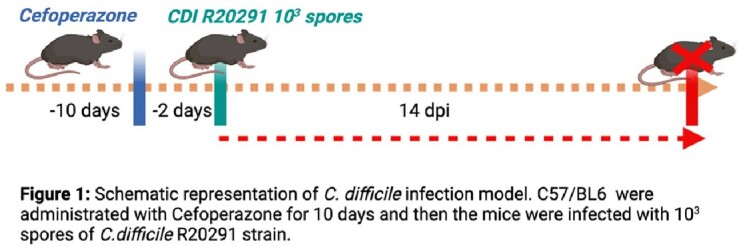

**Results:**

*C. difficile* infected aged mice exhibited greater weight loss at 4 dpi compared to young *C. difficile* infected mice. The gut germinal center response studied at 14 dpi showed mild changes in T follicular helper cells (CXCR5+PD-1+ of CD4+) and germinal center B cells (GL7+CD95+ of CD19+B200+) of young *C. difficile* infected mice, whereas the response was dampened in the aged *C. difficile* infected mice (Figure 2). Further, at 14 dpi ASCs (CD138+CD98+) were seen in the lamina propria of the cecum and colon of the *C. difficile* infected mice, but the frequencies of CD138+CD98+ ASCs as well as IgA+ ASCs were significantly lower in the *C. difficile* infected aged mice compare to young mice (Figure 3).

**Figure:**
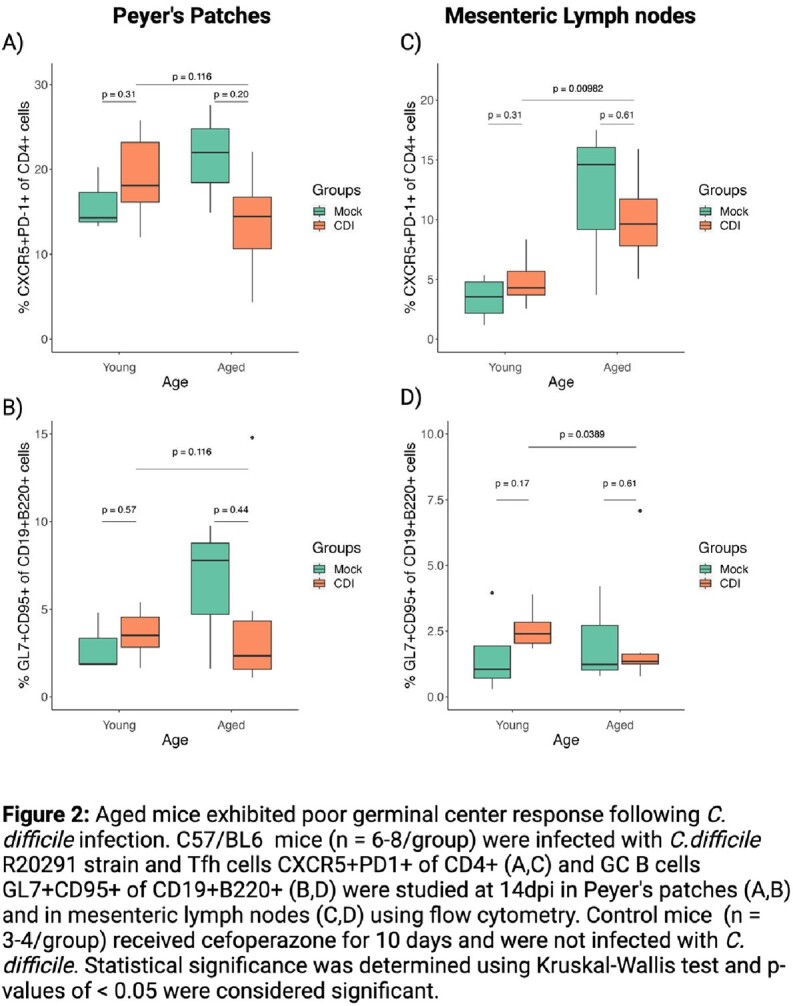

**Figure:**
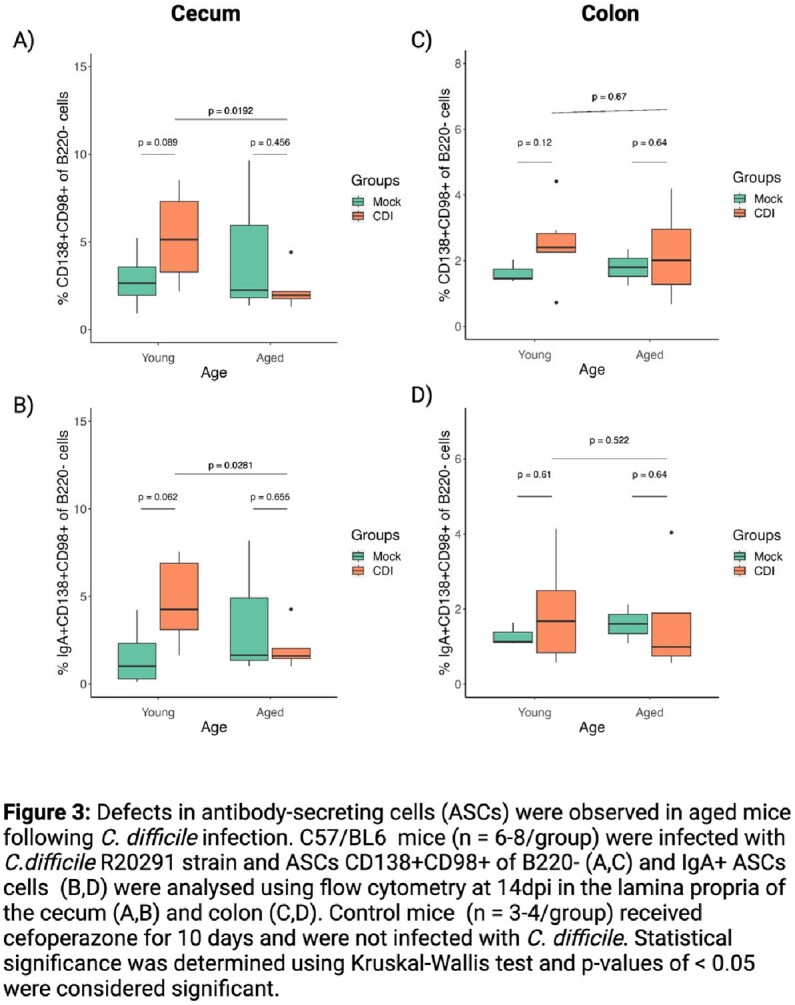

**Conclusion:**

The defect in B cell response during CDI may be responsible for more severe infection and recurrent CDI among aged individual compared to young individuals. It remains to be seen if age-related changes impact antibody production and whether the antibody production during primary CDI provides long-term immunity.

**Disclosures:**

**Vincent B. Young, MD, PhD**, ASM: Senior Editor for mSphere Journal|Debiopharm: Consultant|mSphere: Senior Editor|Vendanta Biosciences: Consultant

